# Using Search Query Surveillance to Monitor Tax Avoidance and Smoking Cessation following the United States' 2009 “SCHIP” Cigarette Tax Increase

**DOI:** 10.1371/journal.pone.0016777

**Published:** 2011-03-16

**Authors:** John W. Ayers, Kurt Ribisl, John S. Brownstein

**Affiliations:** 1 Johns Hopkins Bloomberg School of Public Health, Baltimore, Maryland, United States of America; 2 Center for Behavioral Epidemiology and Community Health, San Diego, California, United States of America; 3 Gillings School of Global Public Health and Lineberger Comprehensive Cancer Center, University of North Carolina, Chapel Hill, North Carolina, United States of America; 4 Harvard Medical School and Children's Hospital Informatics Program, Boston, Massachusetts, United States of America; Institute of Human Virology, United States of America

## Abstract

Smokers can use the web to continue or quit their habit. Online vendors sell reduced or tax-free cigarettes lowering smoking costs, while health advocates use the web to promote cessation. We examined how smokers' tax avoidance and smoking cessation Internet search queries were motivated by the United States' (US) 2009 State Children's Health Insurance Program (SCHIP) federal cigarette excise tax increase and two other state specific tax increases. Google keyword searches among residents in a taxed geography (US or US state) were compared to an untaxed geography (Canada) for two years around each tax increase. Search data were normalized to a relative search volume (RSV) scale, where the highest search proportion was labeled 100 with lesser proportions scaled by how they relatively compared to the highest proportion. Changes in RSV were estimated by comparing means during and after the tax increase to means before the tax increase, across taxed and untaxed geographies. The SCHIP tax was associated with an 11.8% (95% confidence interval [95%CI], 5.7 to 17.9; p<.001) immediate increase in cessation searches; however, searches quickly abated and approximated differences from pre-tax levels in Canada during the months after the tax. Tax avoidance searches increased 27.9% (95%CI, 15.9 to 39.9; p<.001) and 5.3% (95%CI, 3.6 to 7.1; p<.001) during and in the months after the tax compared to Canada, respectively, suggesting avoidance is the more pronounced and durable response. Trends were similar for state-specific tax increases but suggest strong interactive processes across taxes. When the SCHIP tax followed Florida's tax, versus not, it promoted more cessation and avoidance searches. Efforts to combat tax avoidance and increase cessation may be enhanced by using interventions targeted and tailored to smokers' searches. Search query surveillance is a valuable real-time, free and public method, that may be generalized to other behavioral, biological, informational or psychological outcomes manifested online.

## Introduction

About 70% of the 46 million smokers in the United States (US) desire to quit, with 3% of all smokers quitting and staying quit each year [1]. Large decreases in smoking prevalence [2] have, in part, been achieved through stronger tobacco control policies [3]; [4]. Still, smoking remains a leading cause of premature death in the US [5] and globally [6].

Raising cigarette prices, typically via excise tax increases, may be the most effective tobacco control measure [7]; [8]. Higher prices increase smoking cessation, prevent smoking initiation, and reduce the quantity smoked among continuing smokers [9]. However, tax avoidance undermines the public health effectiveness of cigarette taxes [10]. Smokers seek out Internet cigarette vendors because their prices undercut brick and mortar retailers by selling from duty-free zones, low tax jurisdictions, or sovereign Indian reservations [11]–[14].

Although used as a mechanism for tax avoidance, the Internet is also a popular venue for health promotion [15]–[17]. An estimated 4.5% of all Internet searches are believed to be health-related [18] and one in four smokers have sought cessation aid online [19]. Professional internet-based cessation programs appear effective when users are enrolled in their services, although the impact of general information seeking on cessation is unclear [20]–[24].

On April 1, 2009 the US federal cigarette excise tax was increased from $0.39 to $1.01 (USD) per pack under congress' reauthorization of the State Children's Health Insurance Program (SCHIP). Ideally, the “SCHIP tax” would lead to more cessation attempts with limited tax avoidance. However, the price increases may be avoided by buying cigarettes online–thereby undermining cessation attempts. This report presents an early examination of the SCHIP tax's impact on information-seeking behavior in the form of cessation and tax avoidance queries to an online search engine using a real-time, free and public surveillance alternative. Other state specific cigarette excise taxes that occurred near the SCHIP tax were investigated to further validate the methods used for the SCHIP evaluation and consider how taxes interact when occurring in sequence, as they typically do, rather than in isolation.

## Materials and Methods

Traditional surveillance techniques are ill equipped to identify timely temporal and spatial health trends for public health and medical professionals, policy makers, and the public who influence policy choices. An alternative method is to monitor real-time search query behavior [25]–[31]. Google search queries related to smoking cessation, *e.g.* “quit smoking,” and attempts to avoid cigarette taxes, *e.g.* “cheap cigarettes,” were analyzed around the time of the federal SCHIP, and two recent state specific tax increases by comparing across geographies with and without a tax increase. Florida's July 1, 2009 $0.34–1.34 tax increase and New York's June 3, 2008 $1.50–2.75 increase were purposefully selected to demonstrate trends related to state-specific tax increases occurring before (New York) or after (Florida) the SCHIP tax.

Data were obtained from *Google Insights for Search* (www.google.com/insights/search/), a public, free and real-time monitoring of Google search queries. This tool produces relative search volume (RSV) indicators scaled to the highest search proportion week (RSV = 100) with lesser values demonstrating how other proportions compared to the highest proportion. For example, if during the highest search proportion week 400 of every 1,000,000 searches were related to cessation this would be RSV = 100. For another week, if 200 of every 1,000,000 searches were related to cessation this would be RSV = 50, interpreted as 50% of the highest search proportion. RSV indirectly corrects for variation in population size and Internet access, where both are increasing during the study period and would bias any absolute search volume measure.

### Analysis Strategy

Initially a single key term was selected, and then other related terms indicative of the concept were added to form a single composite indicator. For example, “quit smoking” and other related key-terms with strong internal consistency were obtained from Google Insights for Search. The results of this approach are shown in Table S1. Data were not available to independently validate Google methods at the time of this report. However, after omitting terms with unclear motivation, e.g. “smoking cigarettes,” the terms generally had strong face validity and showed strong convergent validity with prior studies [32]. Alternative specifications of the root terms, *e.g.* “stop smoking” for smoking cessation, resulted in a similar sample set of search terms, suggesting the analysis is not dependent on the initial search term selection. Analyses were restricted to the root and next 10 related terms. The reliance on 11 terms for each concept was consistent with prior studies where a few, or even one search term [26], may identify key concepts.

The sample of search terms were used to derive a single RSV trend for their related concept. RSV was estimated from March 2007 through October 2010 for the US, New York, Florida, and Canada, where no similar tax took effect. All estimates, for both avoidance and cessation searches, were on the same weekly RSV scale (0–100), with the highest RSV (100) observed in Florida.

Visual data inspection suggested the likely impact of the SCHIP, and state specific tax increases, was a pulse-effect: an immediate increase in mean RSV around the tax hikes, rather than a change in RSV slope. Therefore, estimates regarding tax impacts were derived from a least squares regression predicting mean RSV during a pre period (about 52 weeks before), implementation period (the week of and two-weeks before and after) and post period (about 52 weeks after each tax increase). Inclusion of an implementation period, instead of a pre-post only model, facilitated estimation of the immediate responsiveness to tax increases while accommodating a washout between the pre and post periods to ensure stable estimates of durable differences between each, since immediate and durable differences in search queries may differ.

A comparison of search behavior within the US before and after the SCHIP tax may be biased due to simultaneous occurring time trends (searches are increasing or decreasing overtime because of another current event, not the SCHIP tax). To control for such bias, all estimates regarding tax impacts were made relative to a control unit where no similar tax took effect during the surveillance period (Canada), to estimate the treatment effect of the tax increase using a quasi-experimental design [33]–[35]. We estimated mean “differences of differences” in RSV, changes in RSV during and after the tax increase in the taxed geography (e.g., the US for the SCHIP tax) compared to changes during and after the tax increase in the untaxed geography (Canada); 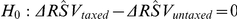. As a result, to the degree thatsearch patterns overlap between the US and Canada independent of tax changes, our strategy controls for simultaneous occurring time trends in search.

Moreover, preliminary analysis suggested cessation searches increased around New Years; as a result, estimates for mean pre-, implementation and post periods were made in absence of New Years effects by including additional indicators in the model for New Years wash-out periods (See Methods S1.)

## Results

**Figure 1:**
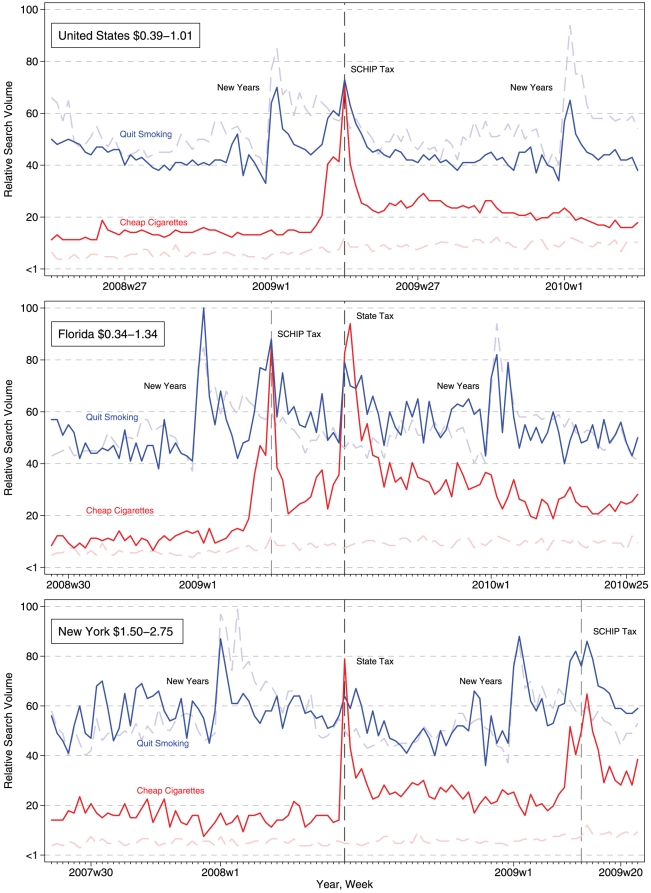
Internet users seek out information on quitting and cheap cigarettes in response to the SCHIP and state specific cigarette tax increases. The vertical reference line indicates the week of the SCHIP and, where applicable, state specific tax increases. The shaded background trends are for the matched control (Canada) where no similar tax took effect.

The $0.39–1.01 federal SCHIP tax increase was associated with immediate sharp peaks for smoking cessation and tax avoidance searches in the US indicative of anticipation of and responses to the tax (**Figure 1A**). In Canada, where no similar tax took effect, there was virtually no change in cessation or tax avoidance searches around the time of or after the SCHIP tax.

**Figure 2:**
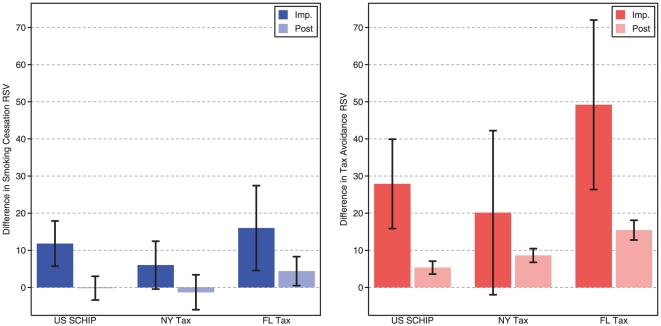
Impact of cigarette taxes on cessation and avoidance during implementation (dark) and post (light) periods. Note: Bars show the mean effect of each tax, and lines indicate the 95% confidence interval, during the implementation period (Imp.) and post (Post) period.

In the US, smoking cessation RSV increased by a factor of 11.8% (95% confidence interval [95%CI], 5.7 to 17.9; p<.001) of the highest RSV comparing differences of differences from the pre-period, weeks 14 through 52 in 2008, with Canada. However, cessation searches quickly abated and approximated the differences from pre-tax levels in Canada during the post period (−0.1; 95%CI, −3.4 to 3.0; p<.904). Tax avoidance searches, on the other hand, increased more and remained higher for longer, than cessation searches. Tax avoidance RSV was 27.9% (95%CI, 15.9 to 39.9; p<.001) and 5.3% (95%CI, 3.6 to 7.1; p<.001) statistically significantly higher during the implementation and post periods, although searches were dissipating in the later portion of the post period (**Figure 2A & 2B**). Moreover, if we extend our analysis backwards to include all related searches since 2004, tax avoidance searches reached an all-time peak in the entire US during the SCHIP tax implementation **[not shown graphically]**.

### State-Specific Models

Changes in search trends for cessation and avoidance stimulated by the state specific tax increases followed a similar pattern as that for the SCHIP tax increase, where taxation typically prompted immediate increases in cessation and tax avoidance searches, with more durable changes for avoidance. The patterns also suggest when presented with two taxes (SCHIP and state specific tax increases), state residents had unique responses to each tax, as indicated by pronounced search peaks during each implementation period compared to Canada where neither tax took effect. Moreover, changes in searches appear to follow a dose-response pattern where larger tax increases produce visually larger increases in avoidance and cessation searches, e.g. in Florida (**Figure 1B & 1C**).

New York's tax increase was associated with a 6.0% (95%CI, −0.4 to 12.5; p<.068) higher cessation RSV during the implementation period compared to differences in searches in Canada; consistent with patterns for the SCHIP tax search trends. The increase in tax avoidance searches was typically twice as large as the increase for cessation searches. Tax avoidance RSV was 20.1% (95%CI, −1.9 to 42.2; p<.074) and 8.6% (95%CI, 6.8 to 10.4; p<.001) higher during the implementation and post periods respectively than changes in Canada (**Figure 2A & 2B**). A related question is: Did the SCHIP tax produce more cessation and avoidance in New York where a state tax had recently elevated the price of cigarettes? The SCHIP, on average, produced more searches for cessation (implementation = 6.4; 95%CI, −1.9 to 14.7, p<.130; post = 7.7; 95%CI, 1.8 to 13.6, p<.007) and tax avoidance (implementation = 3.7; 95%CI, −11.6 to 19.0, p<.631; post = 5.2; 95%CI, 1.7 to 8.6, p<.004) in New York after their state specific tax than overall estimates for the United States **[not shown graphically]**.

The highest RSV observed was in Florida for tax avoidance, higher than RSV for the US, New York, or Canada. Florida's tax increase was associated with a 16.0% (95%CI, 6.8 to 10.4; p<.001) higher cessation RSV during the implementation period. During the post period when searches abated in taxed geographies, Florida's tax increase was associated with a 4.4% (95%CI, 0.5 to 8.2; p<.028) higher cessation RSV for the remainder of 2009 through the 51^st^ week, and may be indicative of the combination of tax increases including the SCHIP tax that recently preceded it. Avoidance RSV was 49.2 (95%CI, 26.4 to 72.0; p<.001) and 15.4 (95%CI, 10.76 to 16.88; p<.001) higher in Florida during the state tax implementation and post period than differences in Canada. It is critical to note, that these represent an *additional increase* over cessation and tax avoidance stemming from the SCHIP tax that preceded Florida's state cigarette tax, suggesting that taxes in close communion, like the 12 weeks between the SCHIP and state tax, have a particularly potent impact upon online behavior.

## Discussion

Smoking cessation and tax avoidance searches peaked around the time the SCHIP tax took effect, suggesting both anticipation of, and responsiveness to, the tax. Smoking cessation searches quickly dissipated to pre-tax levels three weeks after the tax, although, interest in avoiding cigarette taxes almost doubled three weeks to months after the tax, than in the months preceding the tax. These suggest the SCHIP tax was immediately effective at promoting cessation but was more strongly associated with tax avoidance in the longer term. Analysis of two state-specific cigarette tax increases in Florida and New York largely corroborated the SCHIP trends and suggest plausible interaction between tax increases across geographies. For example, the SCHIP tax was associated with larger increases in cessation and avoidance searches in New York, than the overall national estimates, where a recent state tax had already elevated cigarette prices and only 12 weeks after the SCHIP tax Florida's state specific tax increase was associated with unique increases in avoidance and cessation searches over differences from the SCHIP tax.

### Strengths and Limitations

Principally this report demonstrates the potential value of real-time, free and geographically comprehensive search query surveillance for tobacco control, and, more generally, health behavior research. However, understanding the limitations of search query surveillance peculiar to and beyond this study is critical for future research. First, analyses were restricted to ecologic inferences, where specific individuals' smoking behavior could not be induced. Second, search terms related a given concept might change overtime, necessitating sensitivity to such changes. Third, search queries may not capture some tax-motivated outcomes like reduced cigarette consumption that have been associated with cigarette tax increases. Fourth, online smokers may not be representative of all smokers. Smokers who ever used the Internet in 2003 were more likely to have a high school or greater education and have an annual household income in excess of $50K, and were 10 years younger than offline smokers [36]. Internet use has since increased substantially, and differences between online and offline smokers may be minimal, although this needs to be investigated.

Last, the validity of search queries for health behaviors is a major concern. Search queries have strong predictive validity for outbreaks of influenza-like [27]–[30] gastrointestinal [26] and Lyme diseases [31] independent of media coverage. *Google Flu Trends*, for example, provides real-time geographically specific estimates of influenza-like searches in the US, with forecasts eclipsing the timeliness of other modern surveillance (http://www.google.org/flutrends). Moreover, search query trends forecast weekend box-office film revenue, video game sales, the Billboard Hot 100 song chart [37], and unemployment [38]. One validity study suggests that when no other, or only limited, data exist, search queries reveal relevant details about present population behaviors, and provide a useful guide for future population behaviors [37]. As a result, search queries may be valid indicators of smoking related behaviors. Nonetheless, as the correlation with actual behavior is imperfect, the trends described herein should be treated as preliminary, where searches indicate ideation.

### Implications

Given the access to cheaper cigarettes the Internet affords, it may not be surprising that tax avoidance was the more prevalent option for smokers given quitting is a difficult option. It is essential to both minimize tax avoidance by addressing Internet tobacco sales, as governments tax cigarettes to maximize revenue collection, and enhance the impact of taxes on cessation.

About 13 to 23 million searches are conducted each year for tax free, or lower tax, cigarettes with lost government revenue approaching a billion dollars [39]. Moreover, Internet vendors often circumvent other tobacco control policies, like bans on sales to minors [13]. Under the Prevent All Cigarette Trafficking (PACT) Act of 2009 [40], all Internet tobacco vendors are required to verify the age and identity of customers and to pay all applicable taxes. This includes paying taxes for the destination state, which often have higher taxes. US states have new powers under the PACT Act, such as the ability to force out-of-state sellers to collect and remit that state's tobacco excise tax. The implication herein is state tobacco control programs can monitor searches, particularly after a tax hike, and stimulate enforcement against Internet cigarette vendors if avoidance searches spike after the tax hike. US states can also repeat measurements over time to test the impact of their strategies, particularly as state level policies are changing rapidly [41].

Online surveillance can easily lead to targeted and tailored online interventions [42]. Since initial responses to policy changes likely take place on the Internet, it may be appropriate to target Internet users directly. Cobb [43] found only 34% of cessation searches on AOL's Google powered search page were linked to a professional cessation service, suggesting searchers interested in cessation may be sold short to the degree that non-professional services lack evidence-based methods. Regardless access to evidence-based cessation information may be increased through Internet advertising.

Intervention-specific advertisements triggered by users searching for the keyword searches described herein may be purchased from search providers for as little as $0.01 per click. Users linking via these advertisements would be provided evidence-based cessation services. Moreover, avoidance searchers could also be provided a tailored advertisement, *e.g.* “If you want to save money, quitting smoking now saves you the most and is the life saving alternative.” Moreover, such interventions may be tailored to cessation and avoidance searches by considering their stage in the cessation process [44]; [45]. Where cessation searchers are likely contemplating cessation and avoidance searches are pre-contemplative, they should accordingly be provided cues to action including cessation enabling resources and motivation to consider cessation, respectively. The application of online cessation aids could yield enhanced public health effectiveness of cigarette taxation.

More generally, this study advances the use of search query surveillance outside of infectious disease trends, where this approach has largely been developed [25]–[27]; [29]–[31]. Internet saturation is greatest in affluent countries where behaviors, not infectious disease, are the leading cause of premature death [5]. Our work addresses this gap, and accords with a development of a computational social science that draws on digital footprints left in a 21^st^ century society to make scientific inferences [46].

Our strategy may be a useful adjunct to traditional surveillance methods broadly in public health behavior research but may also be a core surveillance method where surveillance advances have been weakly developed. Survey based surveillance of tobacco use and purchasing patterns is very costly, lacks timeliness, and may be biased by strong social desirability –especially when illegal behaviors such as tax avoidance are involved [47]. Archival measures for tobacco use are typically outcome specific. For instance, nicotine replacement therapy sales are not indicative of tax avoidance and neither are calls to a cessation help line [48]–[50]. Moreover, online investigation can usually precede existing surveillance systems and thereby may serve as an early warning system.

Search query surveillance may also be politically relevant. Since policy changes typically require public support [51] evaluation strategies that take years to perform may not provide relevant feedback to public interest groups and voters, thereby limiting public trust in existing, or more reaching, public health policies. Instead, an evaluation that occurs almost immediately after the policy change may inform policy makers and their supporters of the associated costs and benefits when there is still interest to make modifications to, or expand, the policy change. Given the search query data used in this study is publically available, the added transparency may yield robust public health debates among scholars interested in conducting their own analyses and added credibility to lay audiences. Moreover, states and the federal government can use search query surveillance to refine implementation of tax policy and compare searches with other states as benchmarks. They can also use search query surveillance to step-up enforcement of Internet cigarette vendor sales by monitoring search volume while simultaneously monitoring the sales practices of the highest ranked and most visited online cigarette vendors.

### Conclusion

This report demonstrated the potential value of real-time, free and geographically comprehensive search query surveillance for health behavior and health policy research, using some recent tobacco excise tax increases [17]; [25]; [52]. A new research agenda is required to further understand online responses to health policy changes. This agenda may yield ancillary online interventions, thereby, improving the public health effectiveness of public policies. Search query surveillance could, in principle, detect population shifts in health behaviors and outcomes well in advance of traditional methods, especially when these shifts are unanticipated [53]. Moreover, search query surveillance may be generalized to other behavioral, biological, informational or psychological outcomes manifested online, across public health and science.

## Supporting Information

Methods S1 (DOC)

Table S1 **Search terms for smoking cessation and cheap cigarettes.**
(XLSX)
