# Supplementary material for: Using Search Query Surveillance to Monitor Tax Avoidance and Smoking Cessation following the United States' 2009 “SCHIP” Cigarette Tax Increase
Source: PLoS One. 2011 Mar 16;6(3):e16777. doi: 10.1371/journal.pone.0016777 (PMC3059206; doi:10.1371/journal.pone.0016777)
Supplement: Methods S1 — (DOC) [file pone.0016777.s001.doc]

**Supporting Information**

*Selection of the Search Terms*

“Quit smoking” and “cheap cigarettes” were selected as the root terms for identifying search queries related to cessation and tax avoidance **(Table S1)**. The results are displayed in order of their association with the root term with unrelated terms, omitted from the analysis, noted by an asterisk.

*Model Specification (Expanded)*

As described in the text, we estimated mean “differences of differences” in RSV, changes in RSV during and after the tax increase in the taxed geography (e.g., the US for the SCHIP tax) compared to changes during and after the tax increase in the untaxed geography (Canada); . The model may be formally specified as follows:

$$\begin{matrix} Y_{t}=\beta_{0}+\beta_{1}\times implementation period_{t}+\beta_{2}\times post period_{t}+\beta_{3}\times taxed unit_{t} \\ +\beta_{4}\times taxed unit\times implementation period_{t}+\beta_{5}\times taxed unit\times post period_{t}+e_{t} \end{matrix}$$

$\beta_{0}$ estimates the baseline (pre period) average RSV for the untaxed geography (Canada) when all other parameters are set to zero. $\beta_{1}$ and $\beta_{2}$ are estimates for the change in average RSV for the untaxed geography during the implementation and post period each relative to it own mean RSV in the pre period, with implementation and post period indicated by dummy covariates. $\beta_{3}$ estimates the difference in average RSV between the taxed and untaxed geography during the pre period with taxed geography indicated by dummys for a cigarette tax increase. $\beta_{4}$ and $\beta_{5}$ are estimates for the difference in change in average RSV for the taxed geography relative to the untaxed geography, or otherwise are the treatment effects for the tax increase, indicated by an interaction between period and tax dummy indicators [1; 2]. The null hypothesis assumes no difference in mean RSV changes, change for the taxed geography versus change for the untaxed geography, from the pre to implementation and pre to post period. Estimates for differences within the treated units over time are estimated by combinations of parameters, *e.g.* pre period $\beta_{0}$ + $\beta_{3}$, implementation period $\beta_{0}$ + $\beta_{3}$ + $\beta_{1}$ + $\beta_{4}$, and post period $\beta_{0}$ + $\beta_{3}$ + $\beta_{2}$ + $\beta_{5}$.

During the study, pulse-changes in smoking cessation RSV also occurred around New Years--indicative of resolutions to quit smoking. To adjust the model, we included additional breaks in the time groupings including two weeks prior and after New Years week, similar to the implementation period construction, to accommodate these pulse-changes. This allowed us to estimate pre and post period mean RSV in absence of New Year’s effects. In the state-level analyses, where a state excise tax increase is of interest, we also adjusted the model with breaks for the SCHIP tax increase to accommodate a pulse around the time of the US national tax increase.

Time-series data are often autocorrelated, outcomes closer in time tend to be more similar than those farther apart [3], violating the independence assumption for regression residuals [4]. Since our analysis strategy pools times together into 3 mean estimates (pre, implementation and post), and some additional means for New Years periods as appropriate, autocorrelation was not a concern. However, we used a robust variance estimator [5] for hypothesis testing, yielding tests conservative to this and other possible model violations. All tests were two-tailed p<.05.

**References**

1. Aiken LS, West SG, Reno RR (1991) Multiple regression: Testing and interpreting interactions. London: Sage Publications.

2. Brambor T, Clark WR, Golder M (2006) Understanding interaction models: Improving empirical analyses. Political Analysis 14: 63-82.

3. Kirchgassner G, Wolters J (2007) Introduction to modern time series analysis. Berlin: Springer Verlag.

4. Lewis-Beck MS (1994) Applied regression. London: Sage Publications.

5. White H (1982) Maximum likelihood estimation of misspecified models. Econometrica 50: 1-25.
